# A Study of the Interaction between Xanthine Oxidase and Its Inhibitors from *Chrysanthemum morifolium* Using Computational Simulation and Multispectroscopic Methods

**DOI:** 10.3390/metabo13010113

**Published:** 2023-01-09

**Authors:** Sze Ping Wee, Khye Er Loh, Kok Wai Lam, Intan Safinar Ismail

**Affiliations:** 1 Department of Bioscience, Faculty of Applied Sciences, Tunku Abdul Rahman University of Management and Technology, Jalan Genting Kelang, Setapak, Kuala Lumpur 53300, Federal Territory of Kuala Lumpur, Malaysia; 2 Centre for Drug and Herbal Development, Faculty of Pharmacy, Universiti Kebangsaan Malaysia, Kuala Lumpur 50300, Federal Territory of Kuala Lumpur, Malaysia; 3 Natural Medicine and Product Research Laboratory, Institute of Bioscience, Universiti Putra Malaysia (UPM), Serdang 43400, Selangor, Malaysia

**Keywords:** ^1^H NMR-based metabolomics, molecular docking, molecular dynamics, fluorescence quenching, circular dichroism

## Abstract

The current therapeutic approach for gout is through the inhibition of the xanthine oxidase (XO) enzyme. Allopurinol, a clinically used XO inhibitor, causes many side effects. This study aimed to investigate the interaction between XO and inhibitors identified from *Chrysanthemum morifolium* by using computational simulation and multispectroscopic methods. The crude extract, petroleum ether, ethyl acetate (EtOAc), and residual fractions were subjected to an XO inhibitory assay and ^1^H NMR analysis. The EtOAc fraction was shown to be strongly correlated to the XO inhibitory activity by using PLS biplot regression analysis. Kaempferol, apigenin, homovanillic acid, and *trans*-cinnamic acid were suggested to contribute to the XO inhibitory activity. Molecular docking showed that kaempferol and apigenin bound to the active site of XO with their benzopyran moiety sandwiched between Phe914 and Phe1009, interacting with Thr1010 and Arg880 by hydrogen bonding. Kaempferol showed the lowest binding energy in molecular dynamic simulation. The residues that contributed to the binding energy were Glu802, Arg880, Phe 914, and Phe 1009. A fluorescence quenching study showed a combination of static and dynamic quenching for all four inhibitors binding to XO. Circular dichroism spectroscopy revealed that there was no major change in XO conformation after binding with each inhibitor.

## 1. Introduction

Xanthine oxidase (XO) is a homodimer that contains two subunits. It is a member of the molybdenum-protein family since each subunit of the XO contains one flavin adenine dinucleotide (FAD), one molybdenum, and two iron sulfurs (2Fe-2S) (Figure S1). XO consists of three domains, which are the N-terminal, C-terminal, and intermediate domains [1]. XO oxidizes hypoxanthine to xanthine, followed by the formation of uric acid, which is the last product in the metabolism of purine bases [2]. Hyperuricemia leads to the accumulation of uric acid crystals in the joints and kidneys, eventually resulting in gouty arthritis and uric acid nephrolithiasis. The therapeutic approach for gout is by either reducing the production of uric acid through inhibition of XO or elevating the uric acid excretion via renal elimination. The options for anti-hyperuricemic agents are XO inhibitors, uricosuric drugs, or anti-inflammatory agents. An effective treatment for relieving gout is to administer a compound that inhibits the production of uric acid [3]. XO inhibitors are preferable since there are fewer side effects as compared to uricosuric agents [4].

Allopurinol, the structural analog of the natural purine-based hypoxanthine, is the most commonly used XO inhibitor and has been used clinically for more than 30 years [5]. It interferes with the catabolism of purine by inhibiting the activity of XO. However, allopurinol leads to several side effects, including renal toxicity, Stevens-Johnson syndrome, and hypersensitivity syndrome, hence limiting its therapy [4]. Hence, it is crucial to search for a new XO inhibitor with fewer unpleasant side effects. Such active components can be obtained from medicinal plants as a potential source. XO inhibition by phenolic compounds has been demonstrated in several reports. Based on previous investigations, flavonoids are potent inhibitors of the XO enzyme [6]. Apigenin, quercetin, myricetin, and kaempferol have been shown to inhibit XO effectively by occupying the active cavity of the enzyme [7,8]. In addition to their strong effects on XO, flavonoids are abundant secondary metabolites in plants. 

Many plant extracts have been shown to exhibit an anti-hyperuricemic effect by inhibiting XO activity, including *Chrysanthemum morifolium* [9,10]. *C. morifolium* has been commonly used as one of the traditional Chinese medicine prescriptions for gout and pain relief from gout [11]. The flowers are commonly taken as medicinal herbal teas or food supplements [12]. There are various chemical components in *C. morifolium*, such as flavonoids, sesquiterpenes, essential oils, and triterpenes [13]. The wide range of chemical variability and the huge number of metabolites in plants make the processes of detection and characterization challenging to perform [14]. Metabolomics is a powerful tool for identifying and analyzing metabolites for biological properties. The pairing of metabolomics with multivariate data analysis gives a comprehensive view of the data and simplifies the intended analysis. Proton nuclear magnetic resonance (NMR) is commonly employed in metabolomics analysis [15]. With the aid of multivariate data analysis, the simultaneous analysis of all metabolites can be achieved by NMR, despite the huge chemical diversity and extreme complexity of the natural extracts [16].

Molecular docking is an essential aid in computer-assisted drug design and structural molecular biology. The mode of binding of a ligand to a receptor protein is predicted by the ligand-protein docking using a scoring function to sort the docking’s output [17], whereas molecular dynamics (MD) involves the simulation of the dynamic behavior of systems of molecules against time. All the components such as protein, ligand, and explicit (as well as waters), are treated in the simulation box as flexible [18]. MD simulation has been used as a docking-coupled tool based on the induced-fit model, as the stability can be assessed [19], and docking poses can be refined and rescored [20] by the application of MD.

Flavonoids have been extensively studied in previous research in terms of their XO inhibitory activity [7,8]. For instance, kaempferol, 4-hydroxybenzoic acid, and apigenin identified from *C. morifolium* have been found to work additively in XO inhibition by using a metabolomic approach [9]. The ethyl acetate fraction of *C. morifolium*, which is known to contain a large number of phenolic compounds, demonstrates an anti-hyperuricemic effect in rats’ models via non-competitive inhibition of XO [10]. However, there is a lack of studies analyzing the interaction between the XO enzyme and its inhibitors (flavonoids and carboxylic acids) from *C. morifolium* by using a combination of metabolomics, in silico, and in vitro methods. In this study, a different approach was employed to provide additional insights into the inhibition of XO by employing a metabolomic approach in an in silico study in combination with multispectroscopic methods. This approach provides a comprehensive view of the metabolites responsible for the bioactivity of the *C. morifolium* extract. Hence, simplifying the process of identifying important compounds for successive experiments. The protein-ligand interaction was then investigated further by in silico and in vitro studies to give detailed information regarding the inhibitors and proteins in XO inhibition. The information obtained from this study is essential for the development of anti-hyperuricemic drugs in the future.

The main aim of this study is to investigate the activity of XO inhibitors from *C. morifolium* through a sequence of methods including putative identification of XO inhibitors using a metabolomics approach, followed by an in-depth study of protein-ligand interaction using molecular docking, molecular dynamic simulation, and multispectroscopic experiments including fluorescence quenching and circular dichroism to further investigate the interaction as predicted from the simulation.

## 2. Materials and Methods

### 2.1. Chemicals and Reagents

HPLC grade solvents such as methanol, petroleum ether, and ethyl acetate were purchased from ThermoFisher Scientific (Waltham, MA, USA). Dimethyl sulfoxide (DMSO), trimethylsilane (TMS), and deuterated methanol were purchased from Merck (Rahway, NJ, USA). Bovine xanthine oxidase was purchased from Roche Diagnostics (Mannheim, Germany). Potassium phosphate monobasic, di-basic, xanthine, allopurinol, apigenin, kaempferol, homovanillic acid, and *trans*-cinnamic acid were purchased from Sigma Aldrich (St. Louis, MO, USA). Mili-Q^®^ water was used in this research.

### 2.2. Extraction of C. morifolium Flowers

Dried *C. morifolium* flowers were purchased in May 2018 from a local Chinese traditional pharmacy (3.0875° N, 101.6451° E) and sent for plant authentication. The voucher specimen was deposited at the herbarium of the Biodiversity Unit, IBS, UPM, Malaysia (voucher number: SK3139/17). The dried flowers were blended into powder, sieved, and extracted in 80% methanol (1:4 *w*/*v*) by bath sonication (two hours, 25 °C). The crude extract was filtered by vacuum filtration, and the extraction of the plant residues was repeated twice. The crude extracts were pooled and rotary evaporated at 40 °C.

### 2.3. Solvent-Solvent Partitioning

The hydromethanolic crude extract was partitioned four times using petroleum ether with a volume ratio of 3:1 (methanol crude: petroleum ether). All petroleum ether (PE) fractions were pooled and subjected to rotary evaporation at 40 °C, followed by freeze-drying. The defatted crude was partitioned three times using ethyl acetate (EtOAc) and Mili-Q water with a volume ratio of 1:1:3 (defatted crude: deionized water: ethyl acetate). All EtOAc and residual (RS) fractions were pooled separately, rotary evaporated, and freeze-dried.

### 2.4. Identification of C. morifolium Metabolites

The crude extract, PE, EtOAc, and RS fractions of *C. morifolium* were dissolved in deuterated methanol containing 0.03% tetramethylsilane (TMS) at 10 mg/mL. The fractions were analyzed using ^1^H NMR (Varian INOVA 500 MHz, Palo Alto, CA, USA) in the Natural Medicines and Products Research Laboratory, IBS, UPM, Malaysia. The NMR spectra of all fractions were phased, baseline corrected, and binned into 0.04 ppm width (Chenomx NMR suite 5.1 Professional, Edmonton, AB, Canada), followed by pre-saturation (suppression of water peak) at 4.70 to 4.80 ppm. The data matrix output was converted to Microsoft Excel worksheet format, and the output was imported into SIMCA-P Version 13.0 (Umetrics, Umeå, Sweden) for multivariate data analysis. All spectral data were mean-centered, Pareto-scaled, and auto-fitted before partial least squares (PLS) and biplot analyses. A score scatter plot was generated by two principal components, where each point represents an individual NMR spectrum. The metabolite peaks were compared to NMR databases, such as the Human Metabolome Database and the Biological Magnetic Resonance Data Bank, with the aid of Chenomx NMR Suite 7.7.

### 2.5. Xanthine Oxidase (XO) Inhibitory Assay

The xanthine oxidase inhibitory assay was performed using the *C. morifolium* crude, PE, EtOAc, and RS fractions as well as standard inhibitors kaempferol, apigenin, homovanillic acid, and *trans*-cinnamic acid by referring to a previous method [10] with slight modifications. A total of 100 μL of test samples containing dimethyl sulfoxide (100 μg/mL) and 0.1 U/mL xanthine oxidase (100 µL) were added to a 0.05 M potassium phosphate buffer (1.3 mL, pH 7.5). The mixture was then incubated at 25 °C for 10 min. A 1 mL of 0.15 mM xanthine was added into the solution, then incubated at 30 °C for 10 min. The absorbance was measured at 295 nm using a UV/Vis spectrophotometer (Evolution 220, ThermoFisher Scientific, Waltham, MA, USA). The percentage of XO inhibition was calculated as follows:

$$\;\mathrm{Inhibition}\;\left(\%\right)=\frac{{\mathrm{Absorbance}}_{\mathrm{negative}\;\mathrm{control}}-{\mathrm{Absorbance}}_{\mathrm{sample}}}{{\mathrm{Absorbance}}_{\mathrm{negative}\;\mathrm{control}}}\times 100\%$$

Absorbance_negative control_ = Absorbance of the negative control (without inhibitors)

Absorbance_sample_ = Absorbance of the solution containing test samples

A 100 µg/mL of allopurinol was used as the positive control, while DMSO was used as the negative control.

### 2.6. Molecular Docking

#### 2.6.1. Preparation of Protein

XO from the source of bovine was downloaded from the Protein Data Bank (PDB) (ID: 3NVY). The X-ray crystal structure of the XO was complex with quercetin [21]. One of the subunits of the XO homodimer was removed. The small molecules such as quercetin, FAD, and [2Fe-2S] clusters as well as all water molecules were removed. The XO was protonated at pH 7.4. The remaining parameters were maintained at their default values.

#### 2.6.2. Preparation of Ligand

The selected XO inhibitors used in this study were kaempferol, apigenin, homovanillic acid, and *trans*-cinnamic acid. The structures of the four XO inhibitors were generated by ChemDraw Professional 15.0 (PerkinElmer, Waltham, MA, USA) based on the Human Metabolome Database. The ligand was prepared using Discovery Studio, the ligand partial charge method (Momany-Rone), and full minimization.

#### 2.6.3. Molecular Docking Simulation

The molecular docking simulation of the four inhibitors for XO was performed by the CDOCKER program from Discovery Studio Client v16.1.0 (BIOVIA, San Diego, CA, USA) [22]. A site sphere with coordinates 39.0630, 21.8980, and 20.2180 was defined to surround the active site of XO with a radius of 8.2286. The coordinates were defined based on the active site of the crystal structure of XO, consisting of the residues Phe914, Phe1009, Glu802, Leu1014, Leu873, Thr1010, Arg880, and Val1011. A total of 10 poses were obtained for each ligand. The best docking result was determined based on the most negative CDOCKER interaction energy in kcal/mol and the most favorable protein-ligand interaction. The outputs were studied further in molecular dynamics simulations. The validation of the docking method was determined by redocking quercetin on the active site (results are not shown).

### 2.7. Molecular Dynamics Simulation

The best pose from molecular docking simulation for each ligand (kaempferol, apigenin, homovanillic acid, and *trans*-cinnamic acid) was proceeded with molecular dynamics simulation, using Groningen Machine for Chemical Simulations (GROMACS) 5.0.7 (the University of Groningen, The Netherlands) under Linux operating system (Ubuntu 16.04 LTS) [23]. The pre-processing for protein-ligand complexes and ligand files was performed by using CHARMM-GUI (http://www.charmm-gui.org, accessed on 27 August 2018), a web-based graphical user interface (GUI). The molecular systems and input files were generated, and the simulation protocols in CHARMM (Chemistry at Harvard Macromolecular Mechanics) force fields were standardized [24]. The force field selected was CHARMM36. The waterbox size was octahedral with an edge distance of 10.0. Potassium chloride (KCl) ions (0.15 M) were added by the monte-carlo ion-placing method. The whole molecular system was solvated by adding water molecules using the TIP3P water model. The input generator selected was GROMACS, and the temperature was set at 303 K. Energy minimization and equilibration were performed (cooling steps: 5000; cooling target temperature: 300; DSRunInteractive: true; final minimization: full potential; final minimization gradient tolerance: 0; forcefield: CHARMm; grid extension: 8.0; heating steps: 2000; heating target temperature: 700; include electrostatic interactions: true). A constant number of particles, pressure and temperature (NPT) were maintained after system minimization. The production simulations were carried out at 303 K for 10 ns in all the systems. A root-mean-square deviation (RMSD) of protein and ligand was generated. The movement of atoms against time is evaluated by MD using the integration of Newton’s equations of motion as shown below:

$$\frac{d^{2}r_{i}\left(t\right)}{dt^{2}}=\frac{F_{i}\left(t\right)}{m_{i}}$$

where *r_i_*(*t*) is the vector position of the atom *i* at time *t*, *F_i_*(*t*) is the force exerted on atom *i* at time *t*, and *m_i_* is the mass of the atom. Binding energies were calculated using the g_mmpbsa (molecular mechanics Poisson-Boltzmann surface area) method (https://rashmikumari.github.io/g_mmpbsa/single_protein_ligand_binding_energy.html, accessed on 10 September 2018) [24]. The van der Waals energy, binding energy, polar solvation energy, electrostatic energy, and solvent-accessible surface area (SASA) energy of each ligand, as well as the contribution of residues to binding energy, were obtained.

### 2.8. Fluorescence Quenching Assay

The assay was carried out by referring to a previous method [8] with slight modifications. A 600 μL of 4.0 × 10^−7^ mol L^−1^ XO solution was added to the quartz cuvette and followed by titration by successively adding 3 μL of 2.5 × 10^−4^ mol L^−1^ diluted solution of kaempferol, apigenin, homovanillic acid, *trans*-cinnamic acid, and allopurinol separately using a micropipette to produce a range of concentration from 0 to 13.03 × 10^−6^ mol L^−1^. Titrations were performed manually and mixed carefully. The solutions were allowed to equilibrate (5 min) for each addition, then the fluorescence emission was measured at 298 K, 304 K, and 310 K using an Agilent Technologies Cary Eclipse fluorescence spectrophotometer. The excitation wavelength was set at 280 nm, and the excitation and emission slits were 5 nm. The fluorescence background was corrected by subtracting the relevant blanks corresponding to the potassium phosphate buffer (pH 7.5).

### 2.9. Circular Dichroism (CD) Analysis

The analysis was carried out according to a modified method [8]. The CD spectra of XO with increasing concentrations of apigenin, kaempferol, homovanillic acid, and *trans*-cinnamic acid were recorded at 190–260 nm (JASCO J-815 CD spectrometer, JASCO Corporation, Ishikawamachi Hachioji-shi, Tokyo, Japan) using a 1.0 mm quartz cuvette under constant nitrogen flush. The measurement was performed in potassium phosphate buffer (pH 7.5) at room temperature. The concentration of XO was maintained at 1.0 × 10^−6^ mol L^−1^, the molar ratios of XO to kaempferol, apigenin, homovanillic acid, *trans*-cinnamic acid, or allopurinol were varied at 1:0, 1:5, and 1:10. The JASCO SSE (Secondary Structure Estimation) software was used to analyze the CD spectroscopic data with Reed’s reference [25] to obtain the data of various secondary structures of XO with respective ligands.

### 2.10. Statistical Analysis

Statistical analysis was performed using IBM SPSS software, Version 21 (IBM, Chicago, USA). The data were expressed as mean ± standard error of the mean (n = 3). The data were analyzed using one-way analysis of variance (ANOVA), and the significance of the difference between the means was analyzed by Tukey (for equal variances) and Tamhane’s (for non-equal variances) post hoc tests at *p* ≤ 0.05 statistical significance.

## 3. Results and Discussion

### 3.1. Multivariate Data Analysis

**Figure 1:**
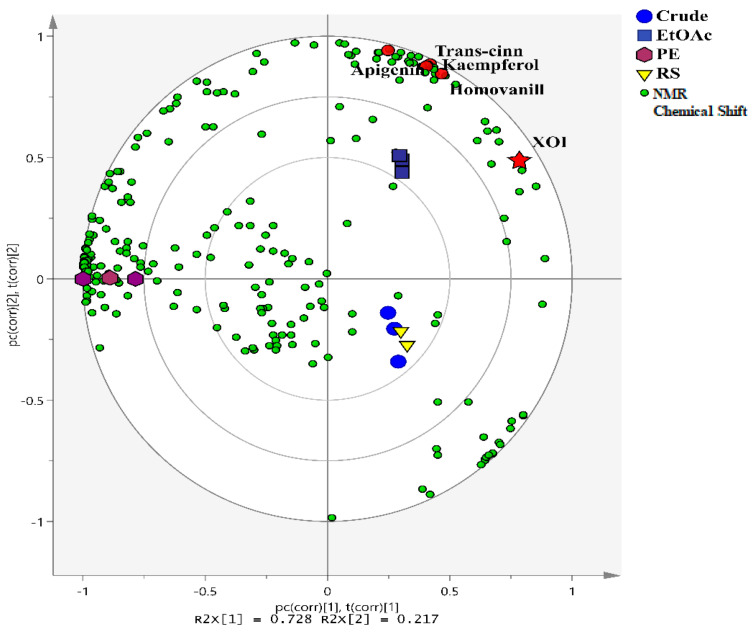
PLS biplot regression analysis describing the relationship between different fractions and XO inhibitory activity. Each point represents an individual NMR chemical shift, red star represents the Y-variable, and the four metabolites responsible for XOI activity are represented by red color circles. EtOAc, ethyl acetate fraction; PE, petroleum ether fraction; RS, residual fraction; XOI, xanthine oxidase inhibitory activity.

In this study, metabolite profiling based on ^1^H NMR spectra was applied to identify the XO inhibitors of *C. morifolium* extract and its fractions. The biplot (Figure 1) showed proximity between the crude and RS fractions, and this cluster showed a clear separation from the EtOAc and PE fractions. The clustering of the crude and RS fractions in the same quadrant indicates that the majority of the metabolites in the crude and RS fractions were almost similar. In other words, the metabolite composition of the EtOAc and PE fractions was distinctive. The distinct distribution of the metabolites in different fractions might be due to their polar nature during solvent-solvent partitioning.

In addition, the correlation between *C. morifolium* metabolites and XO inhibitory activity was also analyzed. The PLS biplot shows that the EtOAc fraction is closely located to the XOI activity (y-variable) in the upper right quadrant of the biplot by PC1. This indicated that EtOAc was strongly correlated with XO inhibitory activity. The metabolites that contributed to the XO inhibitory activity were examined by analyzing the PLS loading plots and the variable importance in projection (VIP) value. Metabolites with significant differences were curated based on their VIP values; chemical shifts with VIP > 1 were considered important for the classification. The error bars of the important variables in the loading plots were also screened to ensure they did not cross the baseline (y = 0). Based on the PLS and VIP values, four metabolites could be important for the XO inhibitory activity, namely kaempferol, apigenin, homovanillic acid, and *trans*-cinnamic acid. Kaempferol and apigenin exhibited an XO inhibitory effect with IC_50_ values of 2.18 ± 0.02 and 3.2 μM, respectively, in the previous studies [8,26]. *Trans*-cinnamic acid was reported to have an IC_50_ of >200 μM according to a study reported by Chang et al. [27], while homovanillic acid is a less potent inhibitor of XO [28]. Hence, these four compounds were selected for successive in silico and in vitro studies.

### 3.2. Molecular Docking Simulation

**Figure 2:**
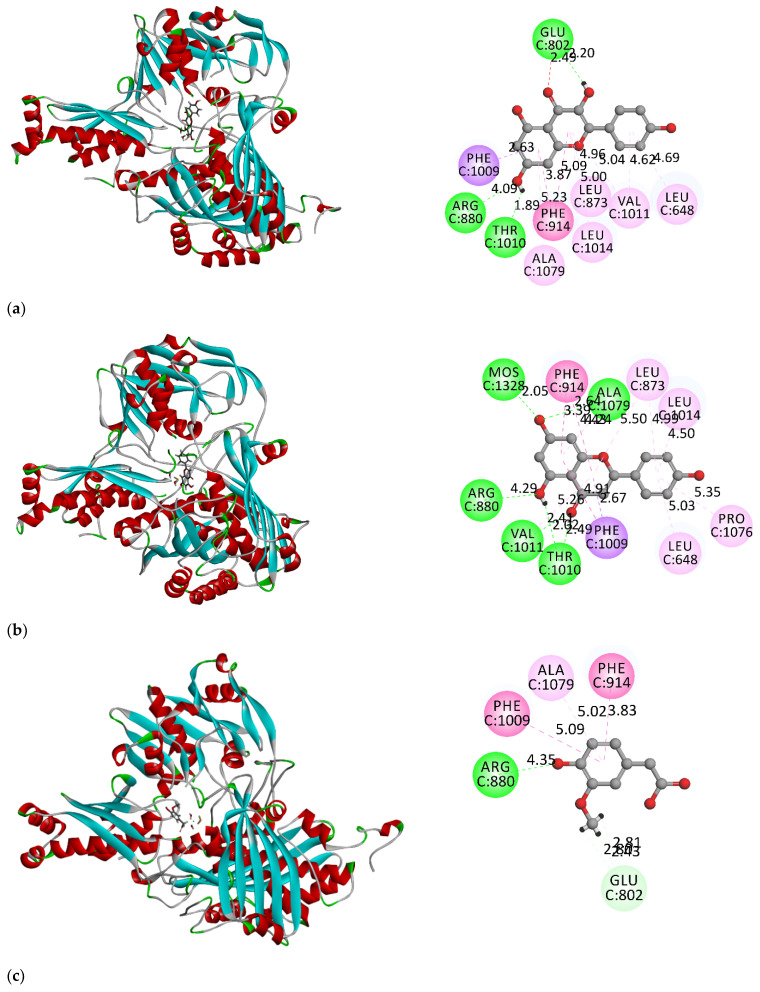

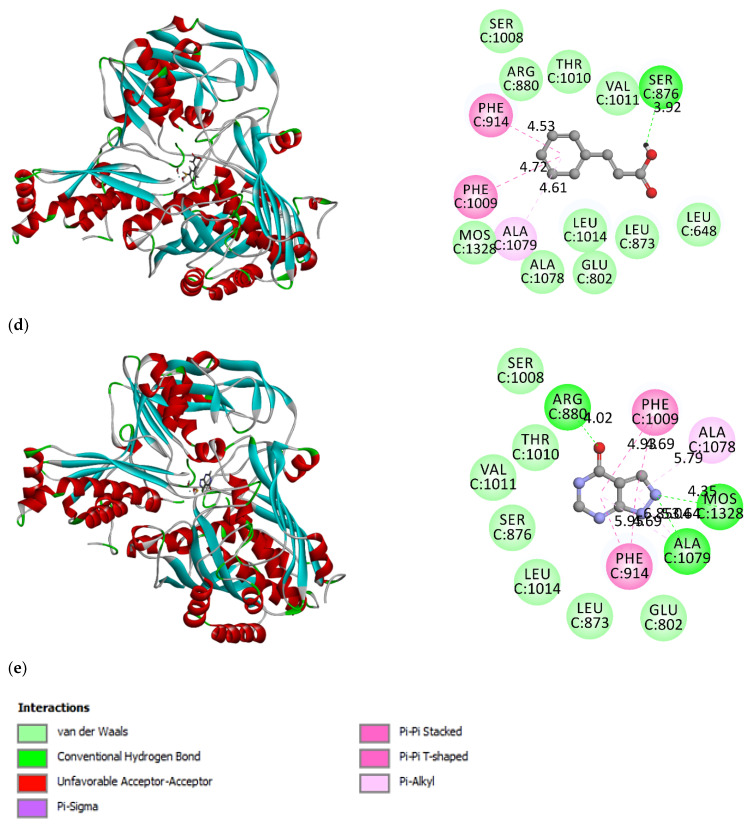
The interactions between (**a**) kaempferol, (**b**) apigenin, (**c**) homovanillic acid, (**d**) *trans*-cinnamic acid, (**e**) allopurinol, and XO residues from molecular docking.

Molecular docking was performed to determine the interaction between XO and the potent inhibitors kaempferol, apigenin, homovanillic acid, and *trans*-cinnamic acid. The outputs are shown in Figure 2. In this study, kaempferol, apigenin, and *trans*-cinnamic acid bound to the binding site and were adjacent to Leu648, Arg880, Phe914, Phe1009, and Thr1010 residues. The C7 hydroxyl group of kaempferol was hydrogen bonded to Arg880 and Thr1010, whereas the C3 hydroxyl and C4 ketone were hydrogen bonded to Glu802 (the chemical structure of kaempferol, apigenin, homovanilic acid, and *trans*-cinnamic acid is provided in Figure S2). In contrast, the ketone group at C4 of apigenin was hydrogen bonded to Thr1010 and Val1011, and the C5 hydroxyl was hydrogen bonded to Thr1010 and Arg880. The C7 hydroxyl group of apigenin also formed hydrogen bonds with Ala1079 and molybdenum. The Phe914 and Phe1009 residues were responsible for the formation of hydrophobic interactions in kaempferol, apigenin, and *trans*-cinnamic acid. In addition, the Ser876 residue was found to interact with the carboxyl group of *trans*-cinnamic acid via hydrogen bonding.

Homovanillic acid is bound to the binding site surrounded by Glu802, Arg880, Phe914, and Phe1009. The residues involved are in accordance with the reported residues in the active cavity [29]. Similar to the other compounds, the Phe914 and Phe1009 residues were also responsible for the pi-pi hydrophobic interactions with homovanillic acid. In addition, a hydrogen bond was found between the 4-hydroxyl group of the homovanillic acid and the Arg880 residue. The results indicated that each compound entered the hydrophobic pocket and hindered the substrate from binding to the active site. Specifically, each compound was inserted into the narrow and long channel of the active cavity, leading to the Mo center of XO. When the active site is bound by inhibitors, it prevents the landing of the substrate and subsequently lowers the catalytic activity of the enzyme.

CDOCKER interaction energy indicates the energy of the non-bonded interaction between a protein and a ligand, where stronger (more negative) CDOCKER interaction energy results in more favorable binding [30]. In this study, both of the compounds, kaempferol (−39.1951 kcal/mol) and apigenin (−36.3686 kcal/mol), had lower CDOCKER interaction energy than allopurinol (−25.3066 kcal/mol), indicating that the binding of kaempferol and apigenin to XO was more favorable than allopurinol. The binding of homovanillic and *trans*-cinnamic acids to XO was moderate, as the CDOCKER interaction energies of both compounds were near those of the positive control allopurinol, which were −26.3666 and −23.6662 kcal/mol, respectively. A molecular docking simulation showed that all four compounds bound to the active site of XO via hydrogen bonds and hydrophobic interactions.

The interactions between the four compounds and XO were correlated to the CDOCKER interaction energy. Kaempferol exhibited strong interactions with most surrounding residues at the binding site of XO, resulting in the most favorable binding with the lowest CDOCKER energy, followed by apigenin, homovanillic acid, and *trans*-cinnamic acid. The structure of the compounds might be one of the factors that affects the binding affinity between the compound and XO. Kaempferol and apigenin are similar to quercetin, which consists of a bicyclic ring moiety and a single ring structure; therefore, more interactions can be formed between the compound and the surrounding residues, increasing the stability of binding. The 3-OH (kaempferol), 5-OH, and 7-OH (kaempferol and apigenin) might be the functional groups that give additional stability to its interaction with the XO enzyme via hydrogen bonding, leading to more favorable binding with stronger interaction energy. As displayed in Figure S3, kaempferol and apigenin are bound into the same active pocket of XO as quercetin with the same disposition and orientation.

Apart from hydrogen bonding, hydrophobic interactions between proteins and ligands also largely contribute to the stability of the binding. The size of the hydrophobic surface is stated to be responsible for the strength of the hydrophobic interaction during complex formation [31]. The benzopyran ring in kaempferol and apigenin contributed to greater hydrophobic interactions with Phe914, Phe1009, Leu873, Leu1014, and Leu648 residues. In contrast, homovanillic and *trans*-cinnamic acids have a much simpler structure with only one phenyl ring. Therefore, less hydrogen bonding and hydrophobic interactions can be formed between the compound and the XO residues, leading to a lower binding affinity. For instance, both homovanillic acid and *trans*-cinnamic acid formed hydrophobic interactions with only three residues, namely Phe914, Phe1009, and Ala1079.

### 3.3. Molecular Dynamics Simulation

**Figure 3:**
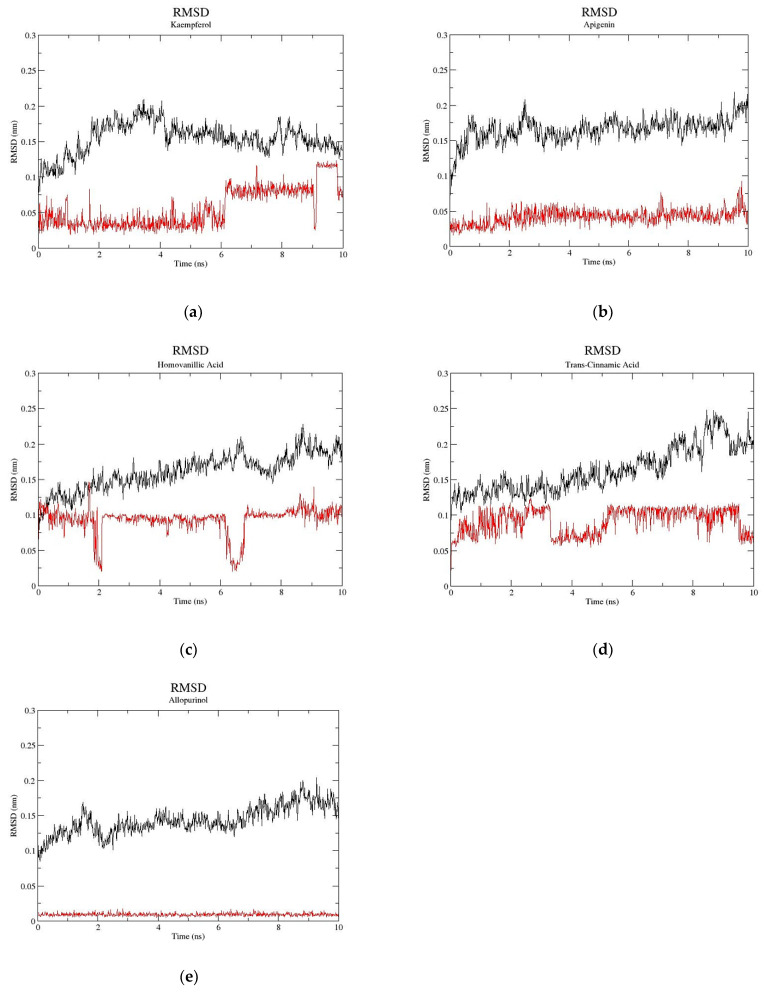
The monitoring of molecular dynamics trajectories. (**a**) kaempferol, (**b**) apigenin, (**c**) homovanillic acid, (**d**) *trans*-cinnamic acid, and (**e**) allopurinol. Black line: the backbone of protein; Red line: respective ligand.

Molecular docking is restricted to a static binding process, where the exact flexibility models assessable to the protein throughout the binding activity are restricted by the docking protocols’ capabilities. Hence, molecular dynamics simulation was performed to further investigate the stability of docked complexes and confirm that molecular docking has produced accurate results. Molecular dynamics simulations for the four compounds kaempferol, apigenin, homovanillic acid, and *trans*-cinnamic acid were performed for a duration of 10 ns. The root-mean-square deviation (RMSD) values of the atoms of the protein backbone and the atoms of the ligand within the binding pocket were monitored to evaluate the dynamic stability of the XO-ligand complexes (Figure 3). The RMSD values reflect the equilibration of each system relative to the initial structures. In this study, after 10 ns, the RMSD of each system for the four compounds tends to be convergent, indicating that the systems were equilibrated. The simulation is considered convergent, and the system has reached equilibrium [32]. Moreover, the RMSD of all four compounds with XO was less than 2 Å, indicating that the deviation in the structural distance between the atoms was small, which reflects higher accuracy and reproducibility in ligand binding. A small deviation of the RMSD value for kaempferol was observed at 6 ns, leading to a smaller RMSD value. This indicated that some changes in the ligand pose occurred at 6 ns and resulted in greater stability of the kaempferol-XO complex. 

The calculation of binding energy was performed using the MM/PBSA (molecular mechanics/Poisson–Boltzmann surface area) method [23] to investigate the interaction between XO and the ligands. The calculated binding energies of the four compounds by the MM/PBSA protocol are shown in Table 1. The compound kaempferol has the lowest binding energy, which indicates the most favorable binding to XO, followed by apigenin, *trans*-cinnamic acid, and homovanillic acid. The results of the MD simulation agreed with those of molecular docking, except that the binding of *trans*-cinnamic acid is slightly more favorable in the MD simulation. Since MD involves the simulation of the dynamic behavior of systems of molecules against time, it has been used as a docking-coupled tool to validate the stability of docking poses [19].

Nevertheless, the binding energy has an unfavorable contribution to allopurinol binding. This agrees with the XO inhibitory activity of allopurinol under physiological conditions. Allopurinol is rapidly metabolized to oxypurinol during its inhibitory reaction, in which oxypurinol has a more favorable binding to XO [33]. Hence, oxypurinol was selected to perform the molecular dynamics simulation and calculation of binding energy using the MM/PBSA method. Oxypurinol showed moderate binding energy, which is less favorable than kaempferol and apigenin but more favorable than *trans*-cinnamic and homovanillic acid. Kaempferol and apigenin were more favorable in binding to XO because of the presence of the benzopyran moiety, which enabled favorable van der Waals interactions with the hydrophobic residues of the XO active site [21].

### 3.4. XO Inhibitory Activity

The XO inhibitors identified from multivariate data analysis of *C. morifolium* fractions were purchased and evaluated for their XO inhibitory activity (Table S1). Kaempferol exhibited the highest XO inhibitory activity (81.87 ± 3.31%) at 100 μg/mL among all other inhibitors. Moreover, it shows no significant difference from the positive control, allopurinol (96.48 ± 2.40%), in this study. Apigenin, which is also a flavonoid, shows lower inhibitory activity (56.60 ± 1.77%) than kaempferol on XO. Kaempferol has been reported to be an effective XO inhibitor because of its C-5 and C-7 hydroxyl groups, which serve a vital role in inhibition [19]. Apigenin, a flavonoid similar to kaempferol, which also consists of C-5 and C-7 hydroxyl groups, however, shows a lower inhibitory effect on XO as compared to kaempferol in this study. This might be due to the presence of three hydroxyl groups in the benzopyran moiety of kaempferol, which are favorable to the electron cloud distribution, contributing to the increase in the electronegativity of kaempferol [6]. In contrast, there are only two hydroxyl groups in the benzopyran moiety of apigenin.

On the other hand, homovanillic acid exhibited the lowest XO inhibitory effect (30.00 ± 2.18%) among the other inhibitors in this study. Homovanillic acid is a less potent inhibitor of XO, according to a study reported by Mohos et al. [28]. *Trans*-cinnamic acid showed moderate inhibition (57.63 ± 6.00%) on XO in the current study. However, it has been reported to be a poor inhibitor of XO, with an IC_50_ > 200 μM [27]. The XO inhibitory assay results agreed with the output predicted from the molecular dynamics simulation, in which kaempferol is the most potent XO inhibitor, followed by apigenin, *trans*-cinnamic acid, and homovanillic acid. The ethyl acetate fraction of the *C. morifolium* extract showed a relatively lower inhibitory effect of 34.92 ± 1.27% on XO as compared to other pure inhibitors. This might be due to the presence of impurities in the fraction except the four identified inhibitors, which results in a relatively lower concentration of each bioactive compound, leading to lower XO inhibition.

### 3.5. Fluorescence Quenching of XO

**Figure 4:**
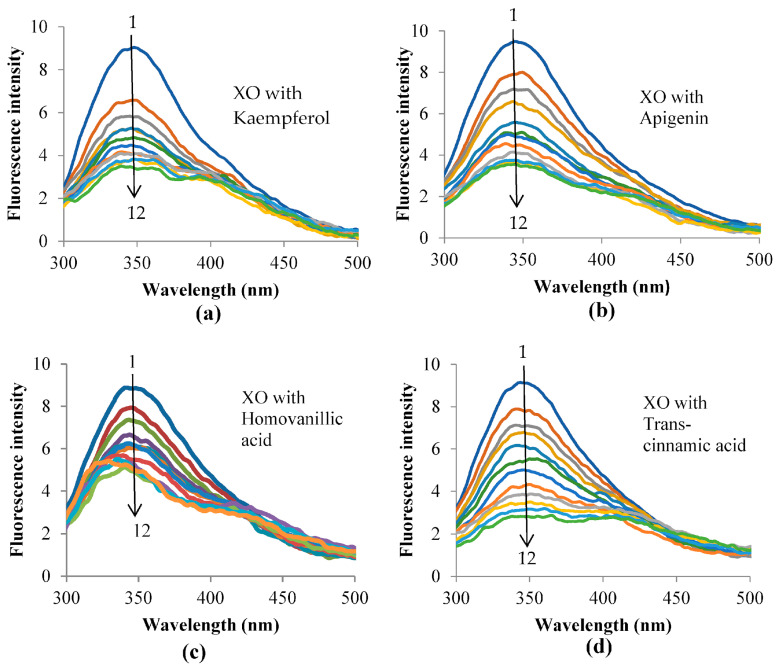
Fluorescence spectra of XO in the absence and presence of (**a**) kaempferol, (**b**) apigenin, (**c**) homovanillic acid, and (**d**) *trans*-cinnamic acid at different concentrations with pH 7.5, T = 298 K, λ_ex_ = 280 nm. The concentration of XO = 4.0 × 10^−7^ mol L^−1^, and the concentrations of the respective inhibitors = 0, 1.24, 2.48, 3.69, 4.90, 6.10, 7.28, 8.45, 9.62, 10.77, 11.90, and 13.03 × 10^−6^ mol L^−1^ for curves 1 (blue) → 12 (green), respectively.

Fluorescence spectroscopy was performed to further characterize the binding between the inhibitors and XO. The fluorescence emission spectra of XO with different concentrations of each inhibitor after excitation at 280 nm are shown in Figure 4. The XO demonstrated a strong emission peak at 342 nm, which was attributed to the XO residues tryptophan and tyrosine [34]. When the amount of the inhibitor was increased, the fluorescence emission peak of XO was reduced regularly and significantly without shifting the position of the emission peak for the reaction of XO with kaempferol, apigenin, and *trans*-cinnamic acid. This indicates that these inhibitors interacted with XO and quenched its intrinsic fluorescence. A blue shift was observed in the XO fluorescence spectra when the concentration of homovanillic acid was increased, and the spectra shifted towards lower wavelengths. This might be due to the movement of tryptophan residues into an interior slit within the XO, which is a more hydrophobic environment [35]. The fluorescence quenching observed in kaempferol, apigenin, and *trans*-cinnamic acid showed that the XO inhibition by the compounds was probably accompanied by collisional quenching, energy transfer, and ground-state complex formation. The blue shift observed in homovanillic acid probably indicated that molecular rearrangements were involved in the inhibition of XO [36].

**Figure 5:**
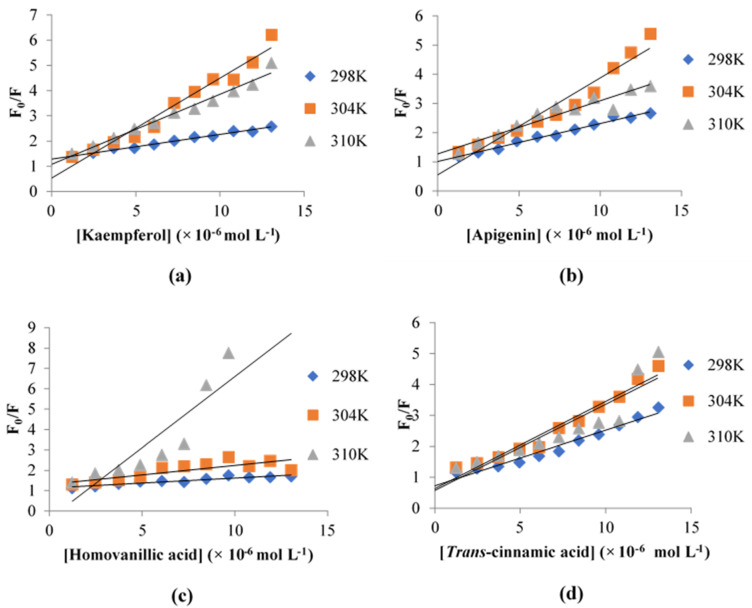
Stern-Volmer plots for fluorescence quenching of XO by (**a**) kaempferol, (**b**) apigenin, (**c**) homovanillic acid, and (**d**) *trans*-cinnamic acid at different temperatures.

To distinguish the mechanism of quenching various inhibitors on XO, the fluorescence quenching output was analyzed by applying the Stern-Volmer equation [34]:

$$\frac{F_{0}}{F}=1+K_{SV}\left[Q\right]=1+K_{q}\tau_{0}\left[Q\right]$$

where *F*_0_ and *F* represent the steady-state fluorescence intensities of XO in the absence and presence of the respective inhibitor. *K_SV_* is the Stern-Volmer quenching constant, determined from the slope of the linear regression of *F*_0_/*F* against the [*Q*] plot. [*Q*] represents the concentration of the inhibitor. *K_q_* is the quenching rate constant of the biomolecule, where *K_q_* = *K_SV_*/*τ*_0_. *τ*_0_ represents the average lifetime of the fluorophore in the absence of a quencher, which is equal to 2.80 × 10^−9^ s [8]. The plots of *F*_0_/*F* against [*Q*] at three different temperatures are shown in Figure 5.

The fluorescence output was further analyzed to acquire the association constant (*K_a_*) by using the modified Stern-Volmer equation [37]:

$$\frac{F_{0}}{F_{0}-F}=\frac{1}{f_{a}K_{a}\left[Q\right]}+\frac{1}{f_{a}}$$

where *f_a_* stands for the fraction of accessible fluorescence. Then, *K_a_* values for the inhibitor-XO complex at three different temperatures were calculated from the linear regression of *F*_0_/(*F*_0_ − *F*) against 1/[*Q*]. The corresponding *K_SV_*, *K_q_*, and *K_a_* values of the respective inhibitors with XO are shown in Table 2.

Generally, high linearity in the Stern-Volmer plot suggests that either a static or dynamic quenching process occurred [38]. From Figure 5, there was no consistent linearity observed in all four inhibitors, suggesting that there was probably a combination of static and dynamic quenching for all inhibitors. From Table 2, the calculated *K_q_* values for all inhibitors at all temperatures were considerably higher than the maximum scatter collision quenching constant of various quenchers with biopolymers, which was 2.0 × 10^10^ L mol^−1^ s^−1^ [39], indicating the quenching was static. However, the *K*_SV_ values for kaempferol, apigenin, and *trans*-cinnamic acid increased when the temperature was increased from 298 to 304 K but decreased when the temperature was at 310 K. In static quenching, the *K_SV_* values tend to decrease with an increase in the temperature, and vice versa for dynamic quenching. Therefore, there might be a combination of both static and dynamic quenching in all inhibitors in this study. Static quenching indicates that the ground-state complex might be formed between XO and the four inhibitors, while dynamic quenching indicates that collision might occur between XO and the inhibitors. Both types of quenching showed that all four inhibitors contributed to XO inhibition. The *K_a_* values for all inhibitors in this study were in the range of 10^5^ L mol^−1^, indicating a moderate affinity between the inhibitors and XO [8].

### 3.6. Circular Dichroism Spectroscopy

**Figure 6:**
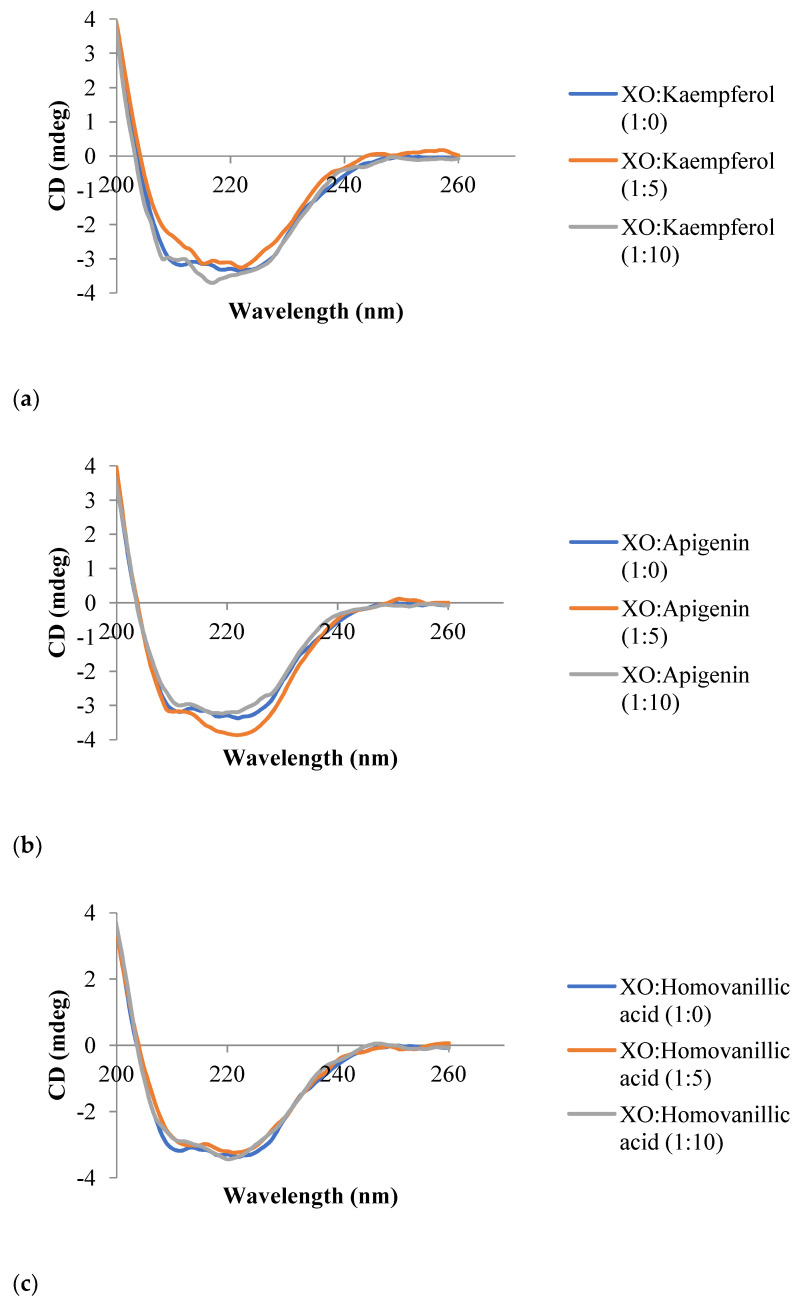

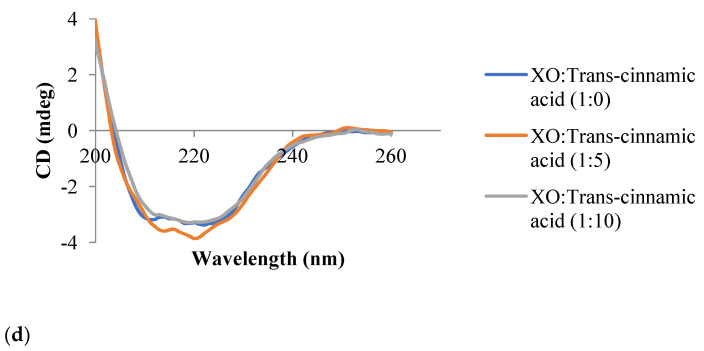
The CD spectra of the inhibitors (**a**) kaempferol, (**b**) apigenin, (**c**) homovanillic acid, and (**d**) *trans*-cinnamic acid with the XO system at pH 7.5. The concentration of XO was 1.0 × 10^−6^ mol L^−1^, and the molar ratios of each inhibitor to XO were 1:0, 1:5, and 1:10.

CD spectroscopy was carried out to investigate the change in conformation caused by the respective inhibitor on XO. The CD spectra of the inhibitor-XO system are shown in Figure 6, while the contents of different secondary structures of XO are shown in Table 3. The spectra demonstrated the characteristic of the *β*-sheet structure as negative bands in the UV region at 216 nm [40]. In general, the increase in the intensity of CD (becomes more negative) is accompanied by the curve shifting away from zero without any significant shift in the position of the peak during an increase in the concentration of the inhibitor, suggesting an increase in the *β*-sheet structure, which predominates the conformation of XO after binding with the inhibitor. [8]. The elevation in the *β*-sheet composition is accompanied by a reduction in *α*-helical structure composition, indicating that the binding of the inhibitor to XO might have destructed its networks of hydrogen bonding. Consequently, the changes caused a minor unfolding of the polypeptides of the enzyme [41].

In this study, the CD intensity for XO (Figure 6) with all inhibitors did not show a significant increase at 216 nm when each inhibitor with different molar ratios was added, indicating there was no major change in the XO conformation after binding with the respective inhibitor. From Table 3, there was a slight increase in the *β*-sheet composition and a decrease in the *α*-helical composition observed when homovanillic acid and *trans*-cinnamic acid bound to XO at a molar ratio of 1:10 compared to XO without the inhibitor (molar ratio at 1:0). Apigenin-XO system showed a slight increase in the *β*-sheet composition and a decrease in the *α*-helical composition at a molar ratio of 1:5, while kaempferol demonstrated an increase in the *β*-sheet composition and a decrease in the *α*-helical composition at both molar ratios of 1:5 and 1:10 when compared to molar ratio 1:0. Kaempferol inhibits XO at both molar ratios of1:5 and 1:10, probably contributing to its high XO inhibitory activity. The unfolding of a hydrophobic cavity with the loss of an *α*-helical content, induced by the inhibitor, possibly results in a decline in the stability of the enzyme; due to the fact that the most stable elements of a protein’s secondary structure are *α*-helices, and therefore leads to a decrease in the XO catalytic activity [8].

## 4. Conclusions

This study employed a combination of approaches, including metabolomics, in silico, and in vitro studies, to rapidly decipher the XO inhibitory activity of compounds in *C. morifolium* extract. The findings provided insights into the inhibition mechanism and interaction of kaempferol, apigenin, homovanillic acid, and *trans*-cinnamic acid identified in *C. morifolium* with the XO enzyme. Kaempferol and apigenin showed strong binding with the XO enzyme among the compounds tested, which indicates their potential to be explored further as a natural anti-hyperuricemic drug. A fluorescence quenching study showed that there was a combination of static and dynamic quenching for all four inhibitors binding to XO. Circular dichroism spectroscopy revealed that there was no major change in XO conformation after binding with each inhibitor. The implementation of the metabolomics approach is highly recommended in drug discovery research as it simplifies the laborious isolation process and provides information regarding the interaction of compounds in biological activities, which the conventional approach cannot provide. Further investigation of the effectiveness of a combination of natural inhibitors in vivo is highly recommended.

## Figures and Tables

**Table 1:** The binding energy between the four ligands and XO is predicted by the MM/PBSA method.

Contribution	Ligand Bound to XO					
	Kaempferol	Apigenin	Homovanillic Acid	*Trans*-Cinnamic Acid	Allopurinol	Oxypurinol
van der Waals energy, ∆*E_vdw_* (kJ/mol)	−148.688 ± 9.848	−150.419 ± 10.214	−88.825 ± 8.976	−60.021 ± 9.090	−80.751 ± 10.305	−93.728 ± 9.469
Electrostatic energy, ∆*E_ele_* (kJ/mol)	−31.383 ± 12.940	−39.748 ± 10.267	−47.019 ± 20.694	−12.039 ± 11.900	−52.820 ± 10.830	−45.434 ± 21.075
Solvent-accessible surface area (SASA) energy, ∆*G*_SA_ (kJ/mol)	−15.812 ± 0.928	−15.576 ± 0.705	−12.120 ± 0.563	−10.185 ± 0.967	−8.452 ± 0.531	−9.248 ± 0.581
Polar solvation energy, ∆*G*_polar_ (kJ/mol)	147.669 ± 22.135	168.773 ± 13.730	134.437 ± 20.071	60.813 ± 21.158	190.432 ± 24.162	124.437 ± 26.528
Binding energy, ∆*G*_bind_ (kJ/mol)	−48.214 ± 17.032	−36.970 ± 15.249	−13.527 ± 14.262	−21.433 ± 12.833	48.409 ± 16.107	−23.974 ± 12.030

**Table 2:** Quenching constant *K_SV_*, quenching rate constant *K_q_*, and modified Stern-Volmer association constant *K_a_* for the interaction between the inhibitors and XO at different temperatures.

Inhibitor	Temperature (K)	*K_SV_* (×10^6^ L mol^−1^)	*K_q_* (×10^14^ L mol^−1^ s^−1^)	*K_a_* (×10^5^ L mol^−1^)
Kaempferol	298	0.0977	0.3489	5.740
	304	0.3964	1.416	2.903
	310	0.2779	0.9925	4.776
Apigenin	298	0.1302	0.4650	1.875
	304	0.3324	1.187	2.855
	310	0.1821	0.6504	2.325
Homovanillic acid	298	0.0491	0.1754	1.783
	304	0.0931	0.3325	3.746
	310	0.6988	2.4957	2.410
*Trans*-cinnamic acid	298	0.1800	0.6429	1.247
	304	0.2823	1.008	2.765
	310	0.2784	0.9943	2.865

**Table 3:** The contents of secondary structures of free XO and inhibitor-XO systems (CD spectra) at pH 7.5.

Inhibitor	Molar Ratio (XO:Inhibitor)	*α*-Helix (%)	*β*-Sheet (%)	Random Coil (%)
Kaempferol	1:0	19.7	65.0	15.4
	1:5	17.1	71.7	11.2
	1:10	0.0	65.8	34.2
Apigenin	1:0	19.7	65.0	15.4
	1:5	10.8	67.0	22.2
	1:10	36.7	63.3	0.0
Homovanillic acid	1:0	19.7	65.0	15.4
	1:5	36.3	63.7	0.0
	1:10	5.1	67.9	27.0
*Trans*-cinnamic acid	1:0	19.7	65.0	15.4
	1:5	36.4	63.6	0.0
	1:10	10.4	66.9	22.7

## Data Availability

All data are available in the manuscript.

## Supplementary Materials

The following supporting information can be downloaded at: https://www.mdpi.com/article/10.3390/metabo13010113/s1, Figure S1: 3D structure of xanthine oxidase. Chain A, B and C are colored in orange, grey and purple respectively. The bound FAD (green), Fe-S cluster (red), MTE (blue) and MOS (yellow) are indicated.; Figure S2: Chemical structure of (a) kaempferol, (b) apigenin, (c) homovanillic acid, and (d) trans-cinnamic acid. Figure S3: The 3D molecular of XO displaying the respective binding of kaempferol (blue), apigenin (yellow), and quercetin (orange) at the active pocket. Table S1: XO inhibitory activity of inhibitors identified from *C. morifolium* extracts.
