# Supplementary material for: A Study of the Interaction between Xanthine Oxidase and Its Inhibitors from Chrysanthemum morifolium Using Computational Simulation and Multispectroscopic Methods
Source: Metabolites. 2023 Jan 9;13(1):113. doi: 10.3390/metabo13010113 (PMC9864848; doi:10.3390/metabo13010113)
Supplement: Supplementary file 1 [file metabolites-13-00113-s001.zip › Supplementary files/Figure S1.pdf]

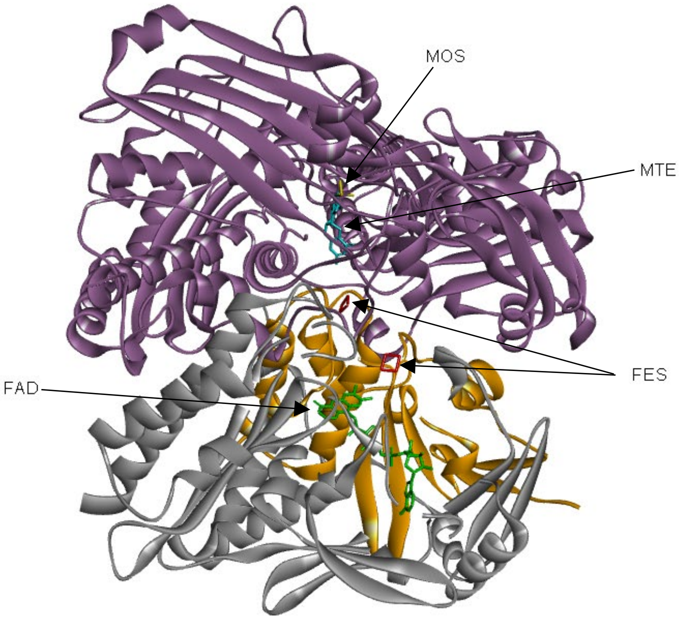

Figure S1. 3D structure of xanthine oxidase. Chain A, B and C are colored in orange, grey and purple respectively. The bound FAD (green), Fe-S cluster (red), MTE (blue) and MOS (yellow) are indicated.
