# Supplementary material for: A Study of the Interaction between Xanthine Oxidase and Its Inhibitors from Chrysanthemum morifolium Using Computational Simulation and Multispectroscopic Methods
Source: Metabolites. 2023 Jan 9;13(1):113. doi: 10.3390/metabo13010113 (PMC9864848; doi:10.3390/metabo13010113)
Supplement: Supplementary file 1 [file metabolites-13-00113-s001.zip › Supplementary files/Figure S3.pdf]

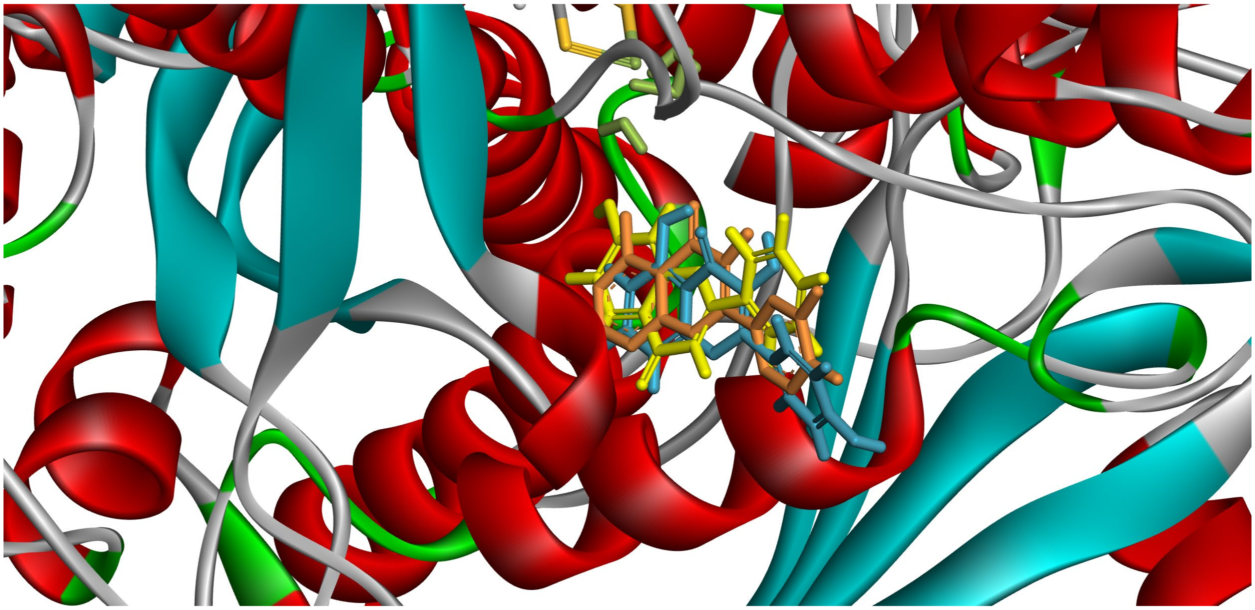

Figure S3. The 3D molecular of XO displaying the respective binding of kaempferol (blue), apigenin (yellow), and quercetin (orange) at the active pocket.
