# Supplementary material for: A Study of the Interaction between Xanthine Oxidase and Its Inhibitors from Chrysanthemum morifolium Using Computational Simulation and Multispectroscopic Methods
Source: Metabolites. 2023 Jan 9;13(1):113. doi: 10.3390/metabo13010113 (PMC9864848; doi:10.3390/metabo13010113)
Supplement: Supplementary file 1 [file metabolites-13-00113-s001.zip › Supplementary files/Table S1.pdf]

Table S1. XO inhibitory activity of inhibitors identified from *C. morifolium* extracts.

**Inhibitor**	**XO inhibition (%)**
Homovanillic acid	30.00 ± 2.18[c]
Trans-cinnamic acid	57.63 ± 6.00[b]
Kaempferol	81.87 ± 3.31[a]
Apigenin	56.60 ± 1.77[b]
Ethyl acetate fraction	34.92 ± 1.27[c]
Allopurinol	96.48 ± 2.40[a]

Values are expressed as mean ± SEM. The data was performed in triplicates (n = 3). Means with different superscript letters show significant difference ($p \leq 0.05$, ANOVA, Tukey HSD's Post Hoc Test). Percentage of inhibition was measured at 100 μg/mL.
